# Supplementary material for: THP-1 Monocytic Cells Are Polarized to More Antitumorigenic Macrophages by Serial Treatment with Phorbol-12-Myristate-13-Acetate and PD98059
Source: Medicina (Kaunas). 2024 Jun 20;60(6):1009. doi: 10.3390/medicina60061009 (PMC11205341; doi:10.3390/medicina60061009)
Supplement: Supplementary file 1 [file medicina-60-01009-s001.zip › Supplementary Figures.pdf]

THP-1 Monocytic Cells Are Polarized to More Antitumorigenic Macrophages by Serial Treatment with Phorbol-12-Myristate-13-Acetate and PD98059

Hantae Jo [1], Eun-Young Lee [1], Hyun Sang Cho [1], Md Abu Rayhan [2], Ahyoung Cho [1], Chang-Suk Chae [1,2] and Hye Jin You [1,2,*]

[1] Cancer Microenvironment Branch, Division of Cancer Biology, Research Institute, National Cancer Center, Goyang 10408, Republic of Korea; jesuswh1@gmail.com (H.J.); eylee@ncc.re.kr (E.-Y.L.); 76030@ncc.re.kr (H.S.C.); 76961@ncc.re.kr (A.C.); csc2022@ncc.re.kr (C.-S.C.)

[2] Department of Cancer Biomedical Science, National Cancer Center-Graduate School of Cancer Science and Policy, National Cancer Center, Goyang 10408, Republic of Korea; 99065@ncc.re.kr

* Correspondence: hjyou@ncc.re.kr

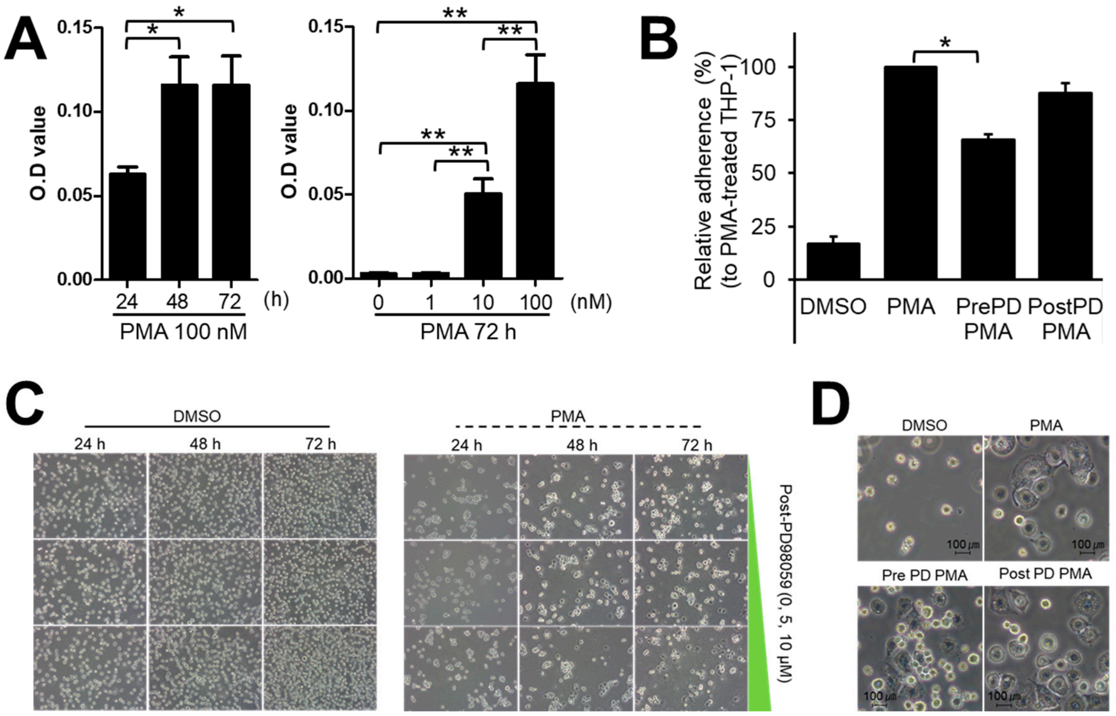

Supplementary Figure S1. **Optimizing conditions for THP-1 differentiation regarding to treatment of PMA or PD98059 were examined by adherence.** (A-D) THP-1 cells were grown on 96 well plates (A, B) or 12 well plates (C, D) for 24 h and treated with 100 nM PMA or DMSO. Adherent THP-1 cells were quantified by MTT assay for PMA-time course (A left) and for PMA-dose response (A right) for the indicated conditions, respectively. (B-D) Some cells were treated with PMA as well as PD98059 together with two different conditions, “PrePD PMA” and “PostPD PMA” as shown in Box and harvested for MTT assay (B), and microscopy (C and D), respectively. (D) Some cells were digitized higher magnification according to scale bars. Data in A and B are means ± SE of at least three independent experiments (*p < 0.01, **p < 0.001). Data in C and D are representative of at least three independent experiments.

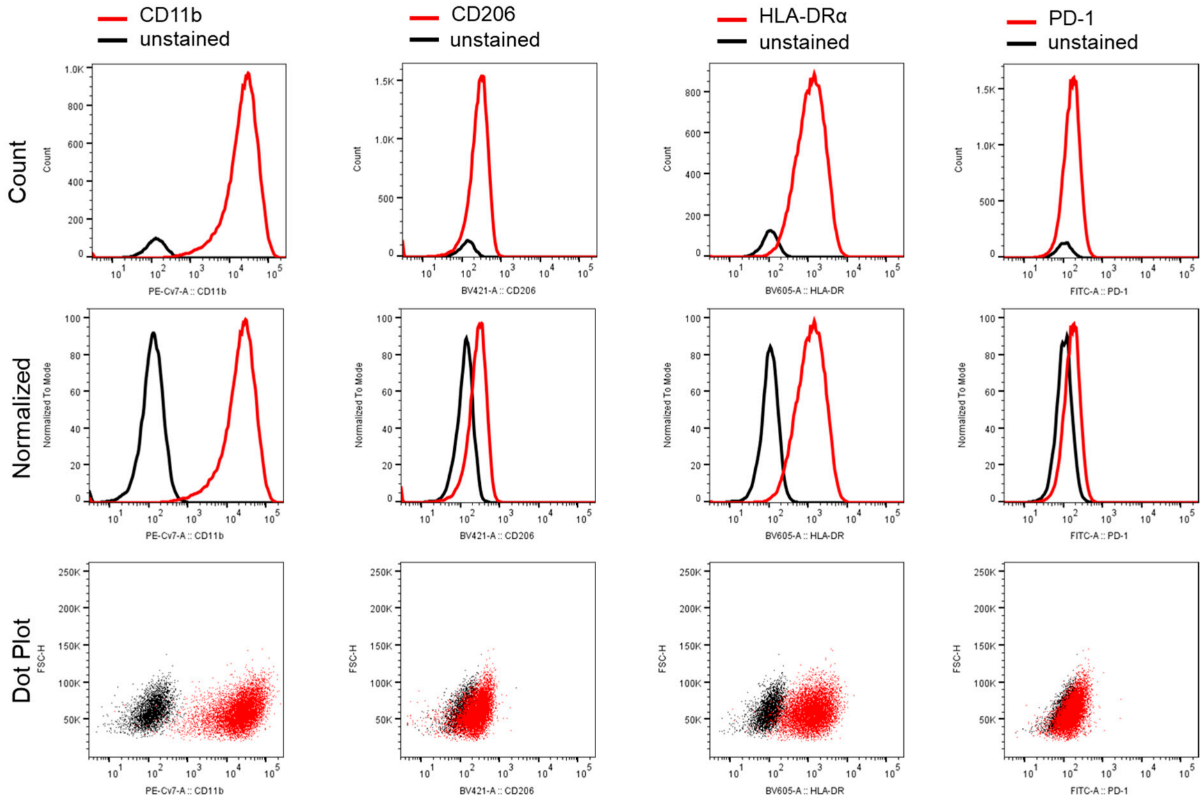

Supplementary Figure S2. Histograms and Dot plots of unstained and stained PMA-differentiated THP-1 cells. THP-1 cells were differentiated in response to 100 nM PMA for 72 h and harvested for flow cytometry. Cells were reacted with fluorochrome-conjugated antibodies against CD11b, HLA-DR, CD206, and CD279 (Supplementary Table ST1) for 30 min at 4 ℃ in the dark while some cells were used without staining for unstained control to gate antibody-specific gating. Each cell subset with a fluorochrome-conjugated antibody (stained, red line or dot) was first evaluated by comparison with unstained cells (black line or dot) and subsequently gated. The histogram of each cell subset with each antibody is shown on the top, the normalized histogram in the middle, and the dot plot at the bottom. This is representative of at least five independent experiments.

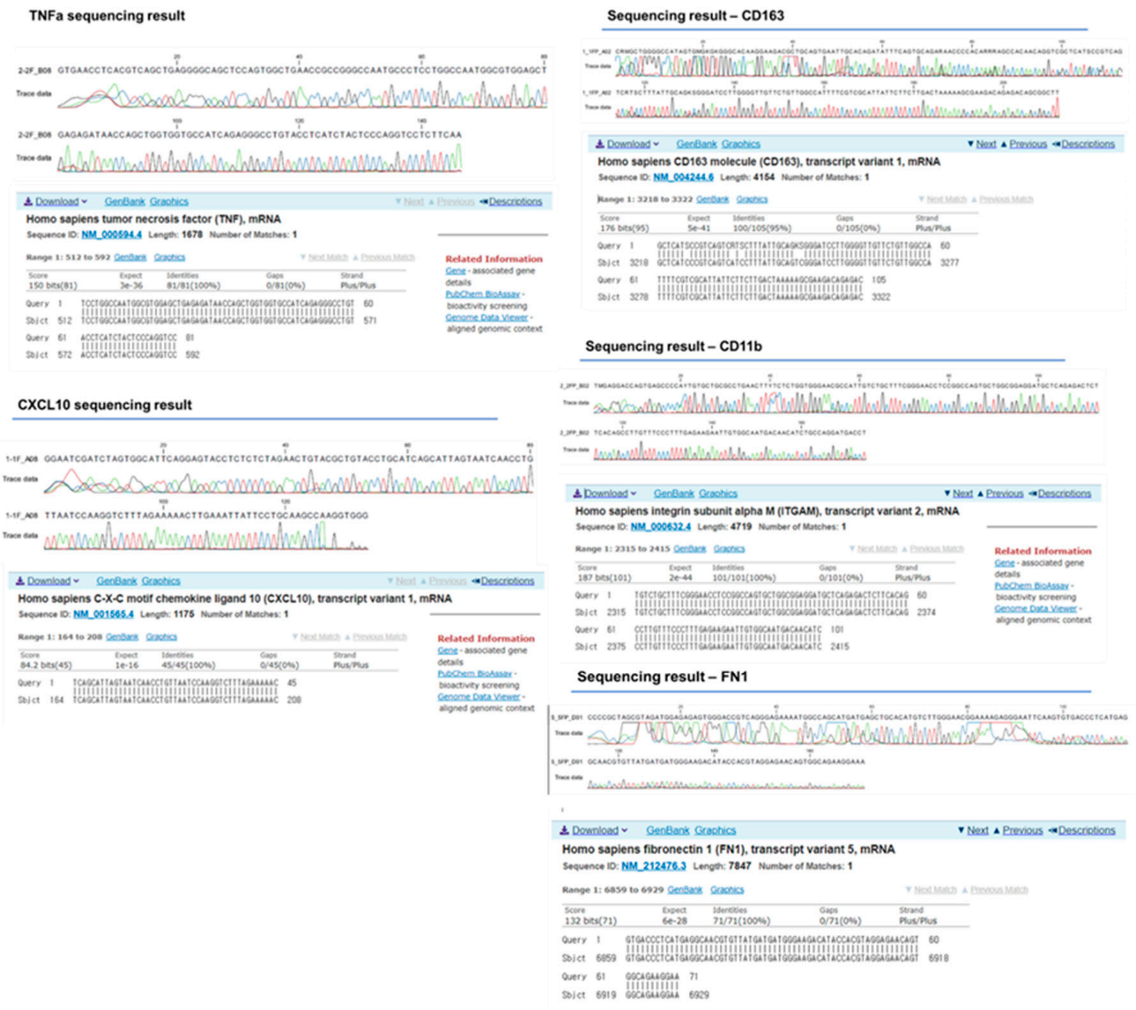

Supplementary Figure S3. Sequencing of RT-PCR products for validation. THP-1 cells were treated with 100 nM PMA or DMSO (vehicle) for 72 h and harvested for RT-PCR. RT-PCR products related to differentiation and polarization were separated by gel electrophoresis and extracted for sequencing analyses. By Sanger methods, sequences were obtained by Macrogen Inc. and aligned in NCBI-BLAST platform, resulting in exact match PCR products to reference genes. Some examples were listed.
