# Supplementary material for: THP-1 Monocytic Cells Are Polarized to More Antitumorigenic Macrophages by Serial Treatment with Phorbol-12-Myristate-13-Acetate and PD98059
Source: Medicina (Kaunas). 2024 Jun 20;60(6):1009. doi: 10.3390/medicina60061009 (PMC11205341; doi:10.3390/medicina60061009)
Supplement: Supplementary file 1 [file medicina-60-01009-s001.zip › Supplementary Tables.pdf]

Supplementary Table S1. Antibodies for flow cytometry

Fluorochrome*	Antibodies	Catalog Number	Clone	Source
FITC	Mouse Anti-Human CD279 (PD-1)	BD 557860	MIH4	BD Pharmingen
PE-Cy7	Mouse anti-Human CD11b	BD 557743	ICRF44	BD Pharmingen
BV421	Mouse anti-human CD206	BD 564062	19.2	BD Horizon
BV605	Mouse anti-human HLA-DR	BD 562845	G46-6	BD Horizon

*FITC: Fluorescein isothiocyanate, PE-Cy7: Phycoerythrin with cyanin-7, BV421: Brilliant Violet 421, BV605: Brilliant Violet 605.

Supplementary Table S2. Primers for RT-PCR

Gene	Forward Primer (5'>3')	Reverse Primer (5'>3')	Accession No.
CD11b	AACTACAGTTGCCGAATTGC	AACTGAAGGTGATGCTGAGG	NM_000632.4
CD36	TTTGGTTCCGTACCCTGTTA	TGCAATACCTGGCTTTTCTC	NM_001001547.3
CD68	GGACTACCAAGAGCCACAAA	GGCATTTCCATGACTAGTGG	NM_001251.3
iNOS	CCCAAGGTCTATGTTCAGGA	GCTCCTCATTCAATTTCAGC	NM_000625.4
HLA-DMα	CACACATTCCTGCACACAGT	CATCCACTCGCAGAACTCTT	NM_006120.4
IFNγ	CAACGCAAAGCAATACATGA	TTACTGGGATGCTCTTCGAC	NM_000619.3
CXCL10	AACTGCCATTCTGATTTGCT	GGCTTGCAGGAATAATTTCA	NM_001565.4
IL6	TGACCCAACCACAAATGCCA	TGAGGTGCCCATGCTACATT	NM_000600.5
TNFα	CGAGTGACAAGCCTGTA	TGAAGAGGACCTGGGAGT	NM_000594.4
CSF1	GCCCCGTTTTAACTCCGTTC	CGCCTCCACCTGTAGAACAA	NM_000757.6
IL10	AAGACCCAGACATCAAGGCG	ACTCATGGCTTTGTAGATGCCT	NM_000572.3
CD163	TCCTTGTGGGATTGTCCTGC	AAGCCGCTGTCTCTGTCTTC	NM_004244.5
CD206	AGGGACGTGGCTGTGGATAA	TCCAAAACCCAGAAGACGCAT	NM_002438.4
TGFβ1	TACATTGACTTCCGCAAGGA	GTGTCCAGGCTCCAAATGTA	NM_000660.7
IL4RA	ACCTGACTTGCACAGAGACG	AGGGCATCTCGGGTTCTACT	NM_000418.4
TGM2	ATCACCAACAACACCGCTGA	CTCCAGGAACACAGGGCTTTA	NM_198951.3
GAPDH	CTGACCTGCCGTCTAGAAAA	GCCAAATTCGTTGTCATACC	NM_002046.7
